# Enhancing communication between dementia care staff and their residents: an arts-inspired intervention

**DOI:** 10.1080/13607863.2019.1590310

**Published:** 2019-03-18

**Authors:** Gill Windle, Katherine Algar-Skaife, Maria Caulfield, Luke Pickering-Jones, John Killick, Hannah Zeilig, Victoria Tischler

**Affiliations:** a Ageing and Dementia@Bangor/DSDCWales, School of Health Sciences, Bangor University, Bangor, Wales;; b Flintshire County Council Social Services, Mold, Wales;; c Dementia Positive, Yorkshire, United Kingdom of Great Britain and Northern Ireland;; d University of the Arts London and University of East Anglia, London, England;; e College of Nursing, Midwifery and Healthcare, University of West London, London, England

**Keywords:** Dementia, arts, training, communication, development, workforce

## Abstract

**Objectives:** The arts are increasingly recognised as important and beneficial activities for people living with dementia. However, there is little peer-reviewed published research exploring arts-based learning for dementia care staff. In response, this paper explores (a) how dementia care staff describe forms of communication in care settings, and (b) the impact on communication following four sessions of ‘Creative Conversations’, an arts-based intervention for skills development.

**Method:** Fourteen care homes received the intervention, delivered as 4 × 2 hour sessions. The intervention uses a range of activities (e.g. poetry, film, music, art making). Twenty–eight care staff were opportunistically sampled (mean age = 42.29), and provided pre-post qualitative data, obtained through interviews. Transcripts were analysed thematically.

**Results:** At baseline, the dominant ‘task-focussed’ nature of care work was described as a barrier to communication, challenging opportunities for developing meaningful relationships with residents. Post-intervention, three primary themes were identified regarding improving communication: (1) *learning through the arts* (secondary themes: *simplicity and subtlety, innovation in communication*, and *strengthening the role of non-verbal communication*), (2) *Enhancing creative approaches to* care (secondary themes: *element of surprise, confidence to experiment* and *catalyst for communication*) and (3) *professional introspection* (secondary themes: *development of empathy, sharing knowledge and experiences* and *a new appreciation*).

**Conclusions:** The intervention validated staff skills and confidence, enabling meaningful interactions that could be creative, ‘in the moment’, spontaneous and improvised. This arts-based intervention, which departs from formal education and fact-based learning may be particularly useful for the development of the dementia care workforce.

## Introduction

In the UK, estimates suggest seventy per cent of people living in residential care homes have dementia or severe memory problems (e.g., Alzheimer’s Society 2018) and the provision of specialist dementia care is now a necessary focus of the care home sector. Legisla"tive changes in Wales, as noted in both the Well-being of Future Generations Act 2015 and the Social Services and Well-being (Wales) Act 2014, demonstrate the Welsh Government’s focus on improving the outcomes, well-being and quality of life of people receiving care services in Wales. Against this background and in response to a commitment for care excellence, this project formed a partnership between a university, a local authority and private sector companies to deliver and evaluate a brief staff development intervention, designed to enrich the quality of the client-career interaction through enhancing communication skills. Recognising challenges around workforce training, this project sought a novel way of enhancing skills through arts-based education.

### Challenges for the dementia care workforce

Care homes face many conflicting pressures involved in delivering day-to-day care, often described as task focussed, and despite best intentions, there is often limited scope for staff and residents to engage in meaningful activities together. This can be a source of tension for the care staff, who feel under pressure to accomplish care tasks, but wish for time to build relationships with residents (Ward et al., 2008). Moreover, the extent to which care staff engage in meaningful interactions is influenced by their communication skills (Ward et al., 2008). Despite the level of complexity around skills for care provision, care work is often perceived as a ‘low skilled’ job, it is often poorly paid, with limited prospects for career progression and professional development (e.g. Adult Social Workforce in England, 2018) and many care staff have no relevant qualification (Clare et al., 2013; Goyder, Orrell, Wenborn, & Spector, 2012; Hussein & Manthorpe, 2012). Consequently, there are considerable challenges for the dementia care workforce, especially as communication in dementia care is not simply about language.

According to the Alzheimer’s Society (2014), communication in dementia care may involve speaking in short and simple sentences, paraphrasing, conveying one question at a time, and when language is difficult, listening, making eye contact, attuning to gestures and body language and reducing environmental distractions (Alzheimer’s Society, 2014). Regardless of the severity of dementia, a person living with the condition may endeavour to find a means to express themselves and communicate to maintain a relationship with their environment (Ward et al., 2008). Here, the ability to communicate moves beyond conversation, and is the means by which people initiate and sustain social interactions.

Interpersonal characteristics of the carer that enable meaningful communication should embed the recognition of personhood and attune to needs (Alsawy et al., 2017). Empathy, taking time over care and foregrounding a person-centred approach underpin the relationship with care home residents, and are predictive of resident mood and less decline in functional status (Anderson, Bird, MacPherson, & Blair, 2016). Such skills are complex and require a certain level of training, yet it is notable that standard training for care home staff often prioritises manual handling, fire safety and safeguarding, rather than developing and enhancing communication skills (Older People’s Commissioner for Wales, 2014; Zeilig et al., 2015). In response, this study sought to develop dementia care education using the arts as the tools for change.

### The arts in dementia care

A review of the involvement of dementia care staff in creative arts programmes indicates that in certain contexts, engaging care staff in creative art activities supports deeper personal connections with residents, enhances understanding of communicative strategies and supports the needs and abilities of residents’ (Broome, Dening, Schneider, & Brooker, 2017). Collectively, these outcomes positively influenced staff-resident interactions and the quality of care practice. Staff who participated in the TimeSlips group storytelling program had more positive views of residents with dementia and devalued residents less compared to the control group staff (Fritsch et al., 2009). A pilot intervention showed that medical students' attitudes, in particular comfort towards dementia, significantly improved following a museum-based arts program designed for people with dementia and their caregivers, and encouraged students to adopt a more humanistic and person oriented approach to care (George, Stuckey & Whitehead, 2013). However, these studies were not designed specifically for staff development and whilst there is a plethora of evidence in grey literature from project reports (e.g. Algar-Skaife, Caulfield, & Woods, 2017; Zeilig, 2016), there is little peer-reviewed published research exploring arts based dementia care learning.

Of particular relevance, Zeilig et al. (2015) developed the Descartes project, an arts-based approach to dementia care staff development, explored in a care home in England. The project explored themes pertinent to dementia care (e.g. language and communication) through various artistic outlets, including poetry, film, and excerpts from plays. The delivery departs from structured and formal learning to the creative exploration of dementia. This proved an engaging educational format for promoting self-reflection and skills recognition, enriching perspectives and understanding of dementia (Zeilig, Poland, Fox, & Killick, 2015). This small pilot study highlights the potential of an arts-based approach for the development of the dementia care workforce.

### Aim

The project sought to build on the work of Zeilig et al. (2015) and address this gap in knowledge through an arts-based staff development intervention ‘Creative Conversations’. Here, the arts are both a mode for delivering the staff development and also tools for supporting and understanding communication. In this sense, the arts are utilised as arts-based education research (Cahnmann-Taylor & Siegesmund, 2018).

This paper qualitatively explores the topic of ‘communication’ (verbal and non-verbal) with care staff in their daily practice. Specifically, it addresses the following questions:

- How do care staff describe forms of ‘communication’ in the care home setting?
- How do care staff describe the influence of the ‘Creative Conversations’ creative sessions on ‘communication’ in the care home setting?

## Methods

### Study design

This project was part of a larger study examining the feasibility and impact of the ‘Creative Conversations’ dementia care training programme. Creative Conversations was a feasibility cluster randomised controlled trial which included observational, quantitative and qualitative measures. This project focuses on the qualitative exploration of ‘communication’, defined as the language and behaviour (verbal and non-verbal) of care professionals within care homes. Pre and post intervention data collection ran from June 2017 to March 2018. Ethical approval was obtained from the lead University’s school ethics committee and the Wales NHS Research Ethics Committee. All participant information sheets and consent forms were provided in both English and Welsh in accordance with Welsh Language Policy.

### Sample selection

Fourteen care homes (local authority and private sector facilities) were recruited to the study within the county of Flintshire, North Wales by the social services team. These were grouped into four clusters (or groups) according to location within the county. Cluster 1–3 each included three care homes and cluster 4 included five homes. Care home staff were opportunistically sampled, based on their availability to commit to the study timescale.

The target sample was 36 ‘front-line’ care staff who interacted with residents on a daily basis. As Creative Conversations was an exploratory study this target number of participants was chosen on pragmatic grounds. Staff unlikely to be regularly present in the care homes over the data collection period (e.g. agency staff) were excluded. Care homes managers were consulted about the study at the outset. The researcher (KAS) then introduced the study to care staff during team meetings. Across the homes, fifty-two care staff were recruited.

### The ‘creative conversations’ programme

‘Creative Conversations’ is an arts-based programme for developing the skills of dementia care staff. It is based on, and inspired by two models that utilise the arts for improving dementia care; the Descartes project (Zeilig et al., 2015) and The Arts and Older People Project at The Courtyard (The Arts and Older People Project & Killick, 2015). At the heart of the programme is a remit for developing compassionate communication and relationship quality, based around the core principles in Table 1. It uses a range of creative activities (poetry, film, music, art making) to engage the emotions, a process that is considered to shape attitude change (e.g. Van Kleef, van den Berg, & Heerdink, 2015). Discussions around topics such as ‘understanding mood changes in people with dementia’ and ‘interpreting the language of people with dementia’ are led by a facilitator.

**Table 1.** Core principles underpinning intervention content and delivery.

Principle:	Example:
**Delivery of the creative sessions**	
The venue(s) is unconventional and confounds expectations	Use of local community venues away from the care home, permitted staff to disengage from their mode of work and relax into a comfortable and neutral environment without risk of distraction
The facilitator's approach is informal	The facilitators blend into the group discussions, offering guidance and sharing perspective when appropriate. Staff are encouraged to take ownership of the discussions, empowering their voices.
The materials booklet is well-designed and attractive, raising expectations	A booklet supports each workshop, containing a collection of poetry, visual arts, song lyrics, pictures, and suggested activities. Music videos are presented via PowerPoint.
All material is available through the facilitator’s website, although access is optional.	Access to an online resource enables staff to review content at any time to refresh their ideas or look back over previous workshop material.
Additional, optional activities are offered outside of the workshops, but no compulsion is exerted.	It is not compulsory for staff to complete extra activities outside the workshops. Instead, the workshops gently encourage curiosity and motivate staff to try activities at appropriate and regular points in their working day.
**Content of the creative sessions**	
No ‘facts’ are given. No instruction is offered. No testing occurs.	The facilitator acknowledges staff as the being the ‘experts in their field ‘and provides them the platform to discuss their work.
All subject matter is of a positive nature. All negative aspects are excluded.	A positive exploration of dementia, where the voices of those living with dementia are prioritised, evolve staffs’ perspective of what it means to live with dementia.
Subject matter is presented for discussion, and no value judgements of responses are made.	Diversity in opinion, perspectives and responses to workshop materials from both the residents and care staff, are celebrated and act as points for discussion and refection.
Activities involve a high degree of sharing.	The first hour of each workshop is dedicated to the sharing of experiences and feedback from trying any new learning from previous workshops.
Creativity is the keynote of all activities both the workshops and optional activities.	The booklet presents materials that facilitate subjective interpretation and the application of imagination. All activities encouraged creativity and innovation in approach/implementation.
Personhood is asserted throughout, both that of the care staff and residents. The message is reinforced at every point without being stated.	During workshop discussions, the unique characteristics, qualities and life experiences of residents’ and care staff are underscored and are used to highlight the wealth of knowledge that can be shared

The programme aims to increase awareness of possibilities and enable the acquisition of practical communication skills to enhance caring relationships. It encourages staff to implement these skills in daily life in the care home (rather than a time-limited activity session, as is often the remit of other arts-based interventions). One example, concerned withverbal language is called ‘Share a Verse’. During the sessions, course members are provided with short, vivid pieces of poetry and each asked to memorise a couple of them to repeat to each other and discuss their meaning and quality. Back in the care home they try them out on residents. When they get a response they repeat the verses on other occasions. This small, uncomplicated activity can increase vocabulary, extend linguistic approaches, and develop a new intimacy between carer and cared-for. In another example, photographs are presented to the group to elicit discussion about the mood and imagery captured within. The group are then given a narrative about the photographs which were taken by a person living with dementia. The narrative is about rediscovering an old hobby and gaining a new perspective after receiving a diagnosis of dementia. Participants are then asked to spend some time taking photographs of anything that appeals to, or intrigues them within the venue setting. The group then share and discuss their thought processes behind the photographs they took. Supporting residents to do this task within the care home, using photography to frame, discover and capture what appeals to residents becomes a suggested activity for care staff to experiment with within their care home. These activities illustrate the arts are both a mode of delivery for the programme as well as a focus of creative skills for care staff to implement outside of the sessions within their care home setting, to enhance communication and interactions with residents.

The intervention period for the main study was 12 weeks. The programme was delivered sequentially to four clusters (or groupings), of care homes, as four two-hourly workshops, described as ‘creative sessions’. Workshop 1 was delivered week 1; workshop 2 in week 2; workshop 3 in week 6 and workshop 4 in week 10. This gap between workshops ensured that staff had time to reflect on the material and to try new approaches and activities. Care staff from each cluster came together for the creative sessions, delivered in two local community venues (a cinema and a pub).

### Data collection

Socio-demographic data were obtained at baseline (Table 2). Qualitative data was obtained through semi-structured interviews, conducted at baseline prior to the start of the training (Time 1), and immediately after intervention completion (Time 2). They took place at one of the two local community venues in Flintshire, Wales. A semi-structured topic guide ensured key questions were asked across the sample, whilst providing the conversational space for issues important to care staff to arise naturally (Table 2). Pre intervention interviews explored staffs’ understanding of and expectations of the intervention and current levels of interaction, both verbal and nonverbal, between care staff and residents. Post-intervention interviews explored staffs’ experience of taking part, the impact this had on their understanding of dementia, their practice and on the residents they worked with. The majority of interviews were conducted by KAS with support from the research team when needed. These were audio recorded and transcribed verbatim by an external service.

**Table 2.** Care staff topic guides.

Baseline	
**What are the words you use to refer to the people you are caring for?** EXPLORE – to start the conversation how do they describe them, are they clients, service users, residents, how are individuals addressed – names, nicknames, and words like ‘dear’ / ‘love’? When are these words used / in which circumstances.	
**What do you talk about with your residents?** EXPLORE – When are conversations based mainly on instructions and when are there opportunities to talk about other things? What sorts of subjects do people want to discuss?	
**What sorts of communication (either verbal or non-verbal) do you most enjoy having with residents?** EXPLORE why? What have you learnt about the people you care for? Have these interactions made you think about things differently? Please use examples.	
**When you are communicating with people who are losing or have lost language, how do you understand what they might need or want?** EXPLORE any evidence of non-verbal strategies to communication, what do the carers recognise from slight nods of the head, small movements of the face and body, repetitiveness of words, etc.	
**We know that your working day is extremely busy and mostly taken up with caring tasks, how much time do you have to spend with residents talking or doing other activities?** EXPLORE whether staff would like more time to engage with residents or whether they feel they have enough.	
**During which care task do you feel you have the most time to communicate / interact with the residents?** EXPLORE what these might be and how they enable closer interaction.	
**How do your residents express themselves?** EXPLORE Language and Behaviour; if they indicate the residents lack communication, explore any evidence of non-verbal strategies to communication, what do the carers recognise from slight nods of the head, small movements of the face and body)	
**Can you tell me something about some of the friendships between residents in your home? How do you support these?** Prompt: by ensuring that people sit with each other at mealtimes / in the lounge? Do similar activities?	
**Follow up**	
**How have you found learning about dementia through film, poetry, and music?** EXPLORE – how did it compare to your first impressions / expectations?	
**How have you found the way your facilitator led the sessions?** EXPLORE – how was it different to other training you have attended? (e.g. John states that he isn’t ‘teaching’ and that there is no right or wrong)	
**Have you had a chance to try any of the activities in practice?** EXPLORE – Was there anything that you found worked very well/ was useful in practice? Tell me your experiences of trying it? In what way did it work very well / was useful?	
**Have the sessions helped you to understand more about how the people you work with feel?** EXPLORE – can you explain in what way? Could you give an example?	
**Has the Creative Conversations made you think more about the way you communicate with the people you work with, especially those who are losing or have lost language?** EXPLORE – can you explain in what way? Could you give an example?	
**Do you think Creative Conversations has made a difference to how you engage with residents? (For example, have you found that you are using more creative arts? How does this compare to how you engaged with residents before you attended the Creative Conversations sessions?**	
**Have you learnt anything new during Creative Conversations?**	
**Do you feel Creative Conversations has made an impact to your day-to-day practice?**	
**Has coming to the Creative Conversations sessions made a difference to the people that you work with?** EXPLORE – in what way? Could you give an example? Has it improved the quality of interaction between yourself and your residents?	
**Do you think Creative Conversations has made a difference to how you engage with residents? (For example, have you found that you are using more creative arts?**	
**How does this compare to how you engaged with residents before you attended the Creative Conversations sessions?** EXPLORE – in what way? Could you give an example?	

In addition to the pre and post interviews, three further focus groups (N = 8, 4, 8) with participating care staff were conducted following the completion of Creative Conversations to explore the lasting impact, feasibility and legacy of the intervention. The focus groups were conducted by MC and a member of the research team and occurred one month after the completion of the programme by the fourth cluster. Recordings of the creative sessions (with participant consent) provided a source of further data for the analysis.

### Data analysis

Descriptive statistics for staff demographics were calculated using IBM SPSS Statistics 22. Qualitative data were managed with EXCEL to allow for anticipated data sharing across the wider project team. The focus group data were combined with the semi-structured interviews for analysis. Prior to data analysis, GW, KAS and MC independently familiarised themselves with four transcripts and met to discuss initial codes and emergent themes. The rest of the data were analyzed by MC using thematic analysis (Braun & Clarke, 2006). This was an iterative process that required the reading, pinpointing, examining and subsequent re- reading of the transcripts. Additional codes were created for data falling outside the coding framework, to ensure important concepts were included. Several meetings between researchers (GW, KAS, MC) permitted greater scrutiny and refinement of themes and sub-themes. An end of project celebration event was arranged for all participants, where emerging themes were presented by KAS. Participants were given the opportunity to discuss whether they felt the themes resonated with their experiences, providing further validation for the findings. For this paper, we provide a narrative overview of the baseline data, and focus the analysis on the post-interviews and session data. Quotes are included to illustrate the identified themes.

## Results

Fifty-two staff were recruited to the main study. All were female, predominantly white British with a lower level of education (see Table 3). Three participants withdrew before the start of the programme. Five participants consented but did not turn up to any workshops. Five participants were not present for the first workshop and fifteen were not present for their last workshop when the pre and post data were collected. Reasons included sickness, annual leave, emergencies within the care home, and leaving their employment. Twenty-four participants provided both pre and post qualitative and demographic data for this sub-study. Additional data from four staff were captured in the focus group. The care staff involved in this study had considerable experience of working in dementia care (M = 11.84 years).

**Table 3.** Demographic information for care staff providing qualitative data (*n* = 28).

**Age, mean (SD)**	42.29	12.33
**Female gender, n (%)**	28	100
**Marital status, n (%)** Married/cohabiting Single Divorced/separated Missing	8 13 6 1	28.6 46.4 21.4 3.6
**Ethnicity, n (%)** White British Missing	27 1	96.4 3.6
**Age leaving FT education, mean (SD)** Missing, (n)	16.71 14	1.73
**Education level, n (%)** Low (No qualifications, 1-4 O levels, NVQ level 1) Medium (5 + O levels, 2+ A levels, NVQ level 2 or 3) High (Higher degree, degree, NVQ level 4 -5) Missing	10 13 4 1	35.7 46.4 14.3 3.6
**Full time employment, n (%)**	15	53.6
**Hours worked per week, n (%)** ≤ 20 21 – 30 31 – 39 ≥ 40	2 15 9 2	7.1 53.6 32.1 7.1
**Length of employment in current care home, n (%)** ≤ 4 years 5- 10 years ≥ 11 years	14 6 8	50.0 21.4 28.6
**Total years employment in care homes, mean (SD)**	11.84	9.20

### How do care staff describe forms of ‘communication’ in the care home setting? a narrative overview of the baseline interviews

This exploration highlights some of the practical communication strategies care staff perceived to be useful, and also some of the tensions experienced regarding task-focussed activities vs meaningful activities. At the outset, most of the care staff described the use of a **‘**language of familiarity and endearment’, to illustrate their perceptions of connections with their residents. They described how nicknames carried sentiment for the residents, and were used to express affection, fondness, and recognised individuality. Terms of endearment, such as *‘duck’, ‘love’* and *‘sweetheart’*, were a subtle way of “*breaking that barrier between you both… making it a bit more personal”*. Care staff appeared to be aware of the need for ‘non-verbal communication’ and described how they used practical tools and techniques, such as flashcards, writing down words or “*rephrasing your question so it’s a yes or no, so he can nod or shake his head”.* In the absence of verbal communication, shared understanding was often reached through a *“process of elimination, guess work”,* and develops over time “*by observing them [residents], to see what their reactions are*”. Care staff acknowledged that often the body language of residents alerted them to their mood, and that meaning can be conveyed through facial expressions: “*you see it in their eyes. They’re looking at you. They’re smiling and they understand*”.CH05

Listening to residents’ stories helped them understand the residents’ identity, and provided greater context to understand their tendencies and behaviours.

*“We used to have a lady who used to wipe the table, she was a dinner lady, so that’s why her behaviour was like that, which helped us to understand.”* BE01

They also highlighted ‘communication tensions’ in knowing how to best approach difficult truths with residents, i.e., concerning the loss of a spouse, or explaining why they could not return to their family home. For care staff new to the role, learning to identify and manage the causes of a resident’s distress proved a frustrating, and at times, an overwhelming experience. Some staff felt pressure from management to always be seen ‘to be doing’ tasks rather than ‘just being’ with their residents. This represented conflict in what constituted ‘meaningful engagement’. *“You have to get certain things done in a day…so I think everyone would like more time to do the little things…and the more social things.”* RH03

Staff reflected that the demands and strain of the system they were operating within (e.g., high staff attrition levels, demanding work load) sometimes imposed a ‘tick box’ approach to their work, producing a tension between their ‘good intentions’, to spend those quality moments with residents and fulfilling their daily duties. This task-focused orientation transpired as a significant barrier in developing meaningful relationships with residents. Although the experience of quality moments with residents were scarce, staff exhibited resourcefulness in identifying and capturing moments with residents. “*Even when we’re sat there doing paperwork, if we’ve got the right staff on, we can talk and write at the same time. So we’re still there with them.”* BE04

### How do ‘creative conversations’ creative sessions influence ‘communication’ in the care home setting?

**Figure 1.**
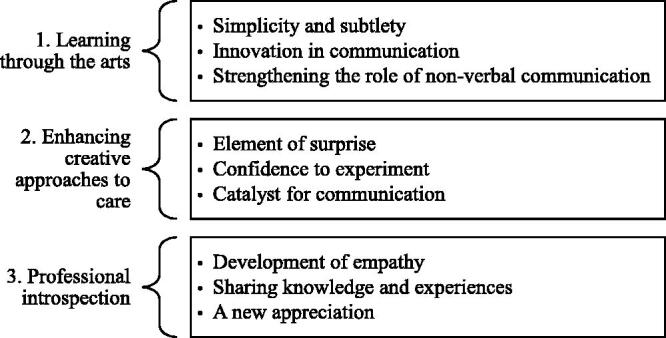
The influence of Creative Conversations staff development sessions.

The follow up interviews explored how the ‘Creative Conversations’ sessions might further influence forms of communication in the care home setting, and revealed three overarching themes with nine sub-themes (see Figure 1).

### Theme one: Learning through the arts

#### Simplicity and subtlety

The workshops prompted discussion about what constitutes a ‘quality interaction’. Staff were encouraged to reflect on how they communicated with their residents and the approaches they took, focusing on the quality of these interaction rather than the quantity.

It appeared empowering for staff to recognise that affection and comfort can be conveyed to residents through simple activities tailored to their needs; pre-planned and elaborate activities were not always necessary to foster an authentic connection.

*It shows you can use anything, doesn’t it? Any little thing. An object, a picture, and it doesn’t always have to be planned either and you can just use that. Even if it’s just for five minutes, it’s still something. It’s still an interaction, isn’t it? They [residents] might not have any interactions all afternoon apart from that* . WD01

Simple activities provoked discussion, curiosity and amusement from residents, opening new channels for communication. Staff reflected on the potential for the more unassuming and subtle activities to be weaved into everyday care tasks, therefore, even a brief encounter with a resident can be made meaningful: “.*‘Cause you think, ‘oh, have I got time?’ But you have got time. And you have made us realise that we can put it in here. You realise how much time you do spend with them [residents].”* CH05

Within the sessions, the exploration of imagery and colours were used to elicit emotion, this led one care staff member to utilise colour within the care home to better understand residents’ perspectives and opinions:

*“We’d had the lift door painted green… and she just started singing ‘’Going Through the Green Door’’…. And then each person I took, I said, “What do you think of the green door?” And they all said something different just because it was a slight change. It was really interesting”.* CH05

#### Innovation in communication

The activities discussed during the workshops built upon staff’s current repertoire of ideas and introduced innovation in their communicative approaches.

“*It’s given me ideas…ideas of what to do in those little situations, just that little bit of quality time, even if it’s not a long time, just a way that you can kind of comfort and just communicate.”* CH06

Importantly, the workshops were a springboard for staff to adapt workshop ideas and stimulated the use of their imagination and creativity. Staff conveyed curiosity to explore novel ways of communicating and reflected on how the activities could be tailored to fit new contexts and to suit individual needs, personalities and preferences. The activities encouraged confidence in their own abilities.

#### Strengthening the role of non-verbal communication

Non-verbal communication was reinforced as a fundamental component in the building of rapport with residents. Inferring meaning from residents’ cues and modes of expression required a high level of self-awareness and curiosity. Encouraging residents to take the lead in the communicative process softened the relational dynamics between staff and residents and allowed feelings of mutuality and equality to arise.

“*Sometimes you don’t have to talk to communicate. You can just sit there and hold someone’s hand. See if they want to have a chat. Let them lead more. Or not say anything, just be there and see where it leads*.” CH07

During a workshop, care staff saw a film of the facilitator interacting with a resident and encouraging her to communicate in her own way and at her own pace. Many staff members commented on this approach and recognised how it diffused the carer-patient dyad: “*It’s just to let them lead. Like [facilitator name] was doing with that resident on the video. He was letting her take the lead*”. CH01

### Theme two: Enhancing creative approaches to care

#### Confidence to experiment

The experimental nature and format of the activities reduced the pressure and expectation on staff to ‘get it right’ or to ‘see a result’. Appreciating dementia through the prism of the arts nurtured a deeper understanding of what constituted as a quality interaction. This allowed staff the confidence and conviction to explore varied forms of communication, for example, through mimicking body language, adjusting one’s tone of voice or body position, through touch or warmth of company. This acknowledgment revived a self-belief in staff that despite their pressured workload, they still hold the capacity to meaningfully and purposively engage with residents, developing a sense of ‘just going with it’. “*Wherever it leads, just go with it*.” WD01

During a workshop, discussions about the power of music and song to engage residents emboldened one staff member to use her own voice to bring joy to the residents in spite of her initial apprehension:

“*I start singing randomly…before, I’d be like, “I’m not gonna do it. I might feel stupid.” But now, you just kinda do it… It doesn’t matter if you’re out of tune because they’re [residents are] enjoying it*”. PH03

#### Element of surprise

Portrayals of dementia through music, poetry and film facilitated a finer appreciation for the role of the arts in the understanding of, and portrayal of dementia. Staff acknowledged how the creative arts opened avenues for residents to channel their emotions and creative capabilities, and remarked how some responses from residents surprised them. Collectively, these observations broadened staff’s existing perceptions of their residents. Reflecting on one of the sessions, one person remarked that “*… it took him to the desert when he was testing missiles ‘cause he was in the forces. So it took him back. He told us all about his army days. He just came alive that day. And I got quite emotional that day with [Resident].”* CH04

#### Catalyst for communication

Staff noted the potential of some activities to encourage social exchanges between residents whom would not normally interact with each other. While engaged in the activities, conversations between residents evolved naturally and led to group discussions, developing a sense of community and a positive atmosphere within the home. In response to one of the activities using fabrics, staff noted: “*we were sort of pushed out because after a while they stopped talking to us and were talking to each other, and then all them were talking and it was really nice to see, because it was four people together who wouldn’t usually talk to each other. “*CH07

Despite the sessions and activities not focussing on reminiscence, the activities supported residents to ‘*go back into their past’* and recall memories from meaningful chapters or events in their lives and share these with others. Staff reflected that the activities supported conversation which led to new knowledge about the person or an insight into their current enjoyments. A more nuanced understanding of residents’ life histories enabled staff to use this knowledge during care practices, for example, as useful conversation prompts, or in deciding upon activities that may be stimulating or relaxing for the residents.

During a workshop staff were encouraged to explore the concepts and meanings within photographs. One staff member later tried this approach with her resident during a routine care task:

“*I was helping a lady into bed and she had a picture of [Café name] and we started talking and she used to run that with her husband and she told me how she met her husband. While she was chatting, I was listening…she was telling me all about her husband. That just led from a photograph on the wall”.* CH05

### Theme three: Professional introspection

#### Development of empathy

Exploration of dementia through the arts was at times an emotional experience. The explorations developed the staff’s empathy, the ability to consider and understand other points of view by becoming more aware of their own values and biases. In reflecting on the use of art activities for exploring dementia, one staff participant noted: “*I like the one where we took photos. It makes you realise things from their point of view. You’d walk out in the garden and you see what you want to see, and it’s not necessarily what they’re seeing…. but you can put yourself in that position where you’re watching*” RH05.

Interactive perspective-building activities with residents helped staff to tune into residents’ viewpoints and daily experiences: *“We had to take people around the home to take photographs… just to see what they looked at. It was really interesting, ‘cause they looked at completely different things…And [Resident] saw really obscure funny, amusing things. Really wacky things. And [Resident], he liked the shed… He was looking at real practical things. But yeah, you kinda learnt a little bit”* CH02. Throughout the workshops, artistic portrayals of people living with dementia proved a moving, and at times cathartic experience for staff. These experiences encouraged staff to develop an understanding of residents through knowledge of their life history, as opposed to viewing them through the narrow window of their diagnosis.

#### Sharing knowledge and experiences

Of particular value to staff was that the workshops brought together staff from different homes with different operating cultures and structures, in a setting away from their place of work. This meant that throughout the workshops there was a sharing of knowledge, and differences in expectations, attitudes and observations were discussed and debated. The discussions reassured staff that they were not isolated in their experiences of caring for vulnerable adults, many of whom are living with dementia, and the complexities, that the role requires. *“It’s nice hearing other people’s stories and how things work where they work when they’re dealing with individuals with dementia and things… You pick up tips that you might not have tried and you think, “That’s a good idea, I might try that with such-and-such” .*PH04

#### A new appreciation

Attending the workshops outside of their place of work provided staff with an alternative environment to pause and reflect on the nature and purpose of their work. Gradually, staff began to appreciate their role in a new light, recognising the value of their work and the expertise that they brought to the caring profession.

“*Some of the things that we’ve seen and done, to a lesser scale, we’ve actually already done. Just not realised. So, what we’ve learnt on top of that now has just made it so much better (.) and interesting.”* WD01

Validation of their person centred approach fostered a sense of pride in their work and provided confirmation that they were *‘on the right route’.*

## Discussion

This qualitative study explored how care staff describe forms of communication in care settings, and how the ‘Creative Conversations’ sessions influenced diverse forms of communication in care settings. The baseline qualitative exploration describes barriers to communication regarding the dominant ‘task focussed’ nature of the work, which challenged the good intentions of care staff for developing meaningful relationships with residents. There are also suggestions of some of the practical communication strategies care staff perceived to be useful, perhaps reflecting their existing experience and time working in dementia care.

Despite care staff’s prior experience at the outset of the study, the different approaches to care facilitated by the arts intervention further enriched their knowledge and skills for communication. It enabled staff to see that communication in dementia as something that is full of variation (gestural, verbal, silent) and responsive to diverse needs. It supports the core principles of person centred care, enabling people living with dementia ‘to be taken seriously and to be understood’ and ‘to be accepted and respected as they are’ (Von Kutzleben, Schmid, Halek, Holle, & Bartholomeyczik, 2012; Van Der Roest, Meiland, Maroccini, Comijs, Jonker, & Dröes, 2007).

A broader aspect connected with the validation of existing skills is apparent. The analysis suggests that the original findings of Zeilig et al. (2015) for promoting self-reflection and skills recognition were established in this study. The opportunity for reflection of care practice and encouragement to enhance their communication skills enabled staff to embed the arts-inspired skills into daily care tasks. A clear recommendation for practice is that this arts-based staff development intervention confirms the importance of finding quality moments in ‘task focussed’ activities.

This is especially important for the person living with dementia, as their former connections with friends and families, who knew them well, are now largely replaced with care staff interactions, who may know little about the person (Anderson, Bird, MacPherson, & Blair, 2016). Family and care professionals often report communication difficulties to be stressful and anxiety provoking, with miscommunication increasing distress for both the person with dementia and the carer (Vandrevala, Samsi, Rose, Adenrele, Barnes, & Manthorpe, 2017; Dooley, Bailey, & McCabe, 2015). As care staff may view the behaviour of a person living with dementia as characteristic of the condition itself, rather than as a symptom of possible unmet needs or an attempt to communicate (Vandrevala, Samsi, Rose, Adenrele, Barnes, & Manthorpe, 2017) interventions such as Creative Conversations, which help care staff understand how people with dementia endeavour to communicate, are critical to good quality care.

Perhaps what distinguishes Creative Conversations from others is its pedagogical approach to teaching and learning (Table 1), e.g. no facts are given and no judgment is passed. Care staff are positioned as experts. The depictions of dementia through various art forms and from multiple perspectives enables care staff to explore their emotional reactions to dementia and attune to the perceptual experiences and communicative cues of people with dementia. The programme also supports reflective group discussions, which are increasingly recognised as an important and effective learning format within dementia care training (Law, Patterson, & Muers, 2017; Morris, Horne, McEvoy, & Williamson, 2017; Surr et al., 2017). However, there is little in the way of theory to suggest whether the arts-based elements can be more influential than reflective discussions without arts for care staff. There are many reasons for this absence of theory, including insufficient theorisation of the whole field of arts and dementia care (e.g. Windle et al., 2018). Given that the arts in education may be particularly useful in prompting opportunities for fresh discussions (Cahnmann-Taylor & Siegesmund, 2018), a possible avenue for future research could compare between creative sessions and traditional opportunities for reflective practice.

Perhaps one of the most valuable aspects of the intervention was the opportunity for care staff to communicate with others from different organisations and to do this out of the care home setting, in an environment not traditionally used for educational purposes. This contrasts with the original Descartes project which was delivered in the care home, and general dementia care training, which is usually delivered ‘on site’ (Eggenberger, Heimerl, & Bennett, 2013).

Moving beyond the professional dementia care workforce, estimates suggest there are over 670,000 unpaid carers in the UK (Alzheimer's Society, 2014). Within this demographic, there is a lack of research exploring the communicative challenges of family carers, and how these are differentially perceived and addressed between partners and offspring carers, whose experiences of caregiving is markedly different (Chappell, Dujela, & Smith, 2015; McCabe, You, & Tatangelo, 2016; Tatangelo, McCabe, Macleod, & You, 2018). Creative Conversations may hold broader potential to support family caregivers, which further research could investigate.

### Strengths and limitations

A major strength of this study was the partnership from the outset with the social services department within a local authority, who were invested in care quality improvement. Fourteen care homes were successfully recruited, and we can be fairly confident in suggesting this would not have happened without this partnership. However, as with most research studies, we had an ‘opt in’ procedure for the staff who took part in the research, which may have skewed the study sample. Although anecdotal, the research team involved in data collection and intervention delivery felt that perhaps the staff (and care facilities) that could have benefited the most were the least vocal in the sessions, were absent from some of the sessions and didn’t always undertake the suggested activities to practice before the next sessions. In contrast, the facilities that had a culture of continuous improvement, valued the quality of their relationships with residents, and whose management strongly supported their staff’s professional development may have benefited more from the programme. These organisational factors may ultimately impact on the future impact and sustainability of Creative Conversations.

This study failed to obtain data from twenty participants, due to their absence from the workshops that coincided with data collection. Unfortunately it was beyond the resources available in this study to pursue these participants. The demographic data show the average time for the participants in their caring role was eleven years. This length of service is reflected in the baseline interviews, which highlight the experience of the staff. This was not an intention on the part of study recruitment and further research could look at restricting the participant eligibility to those more recent to the role. Given the impact of the intervention on the communication skills of those with considerable experience, it is reasonable to assume that the intervention could have a greater impact on those with less experience. Further research could also usefully explore the impact of the programme for male care staff, as all the participants in this study were female.

The qualitative approach adopted in this study potentially captures deeper meaning that could be over-looked by standardised measurement tools, however this is dependent on participants recalling feelings and details that are reliant on memory and subject to interpretation bias. Unfortunately, it was beyond the resources of this study to formally collect qualitative data during the delivery of the programme (between each creative session) that might have elicited more in-depth information about how care staff may have utilised and put into practice some of the new ideas and approaches. The method of structured observation offers a way of quantitatively capturing ‘in the moment’ reactions that might otherwise prove difficult to articulate. Forthcoming findings from the main feasibility study of Creative Conversations (that were beyond the scope of this project) focusses on quantitative outcomes and observations of the resident and carer interactions, and will augment the findings of this qualitative exploration.

## Conclusion

Caring for people living with dementia can be emotionally and physically challenging, and without the appropriate support and training staff are at risk of burn out (Pitfield, Shahriyarmolki, & Livingston, 2011). Collectively, these factors pose serious threats to the consistency and quality of care for older vulnerable adults. Our findings suggest the arts-based approach and underlying principles of the Creative Conversations intervention, which moves away from formal education and fact-based learning to a remit for developing compassionate communication and relationship quality, may be particularly useful for the development of the dementia care workforce. It positions care staff as experts with much to offer, and so validates their role. It encourages spontaneity and creativity on the small scale, and facilitates an understanding that creative interactions can be ‘in the moment’ and improvised. It illustrates the potential for enriching the experiences of staff, and therefore their residents. Further research comparing Creative Conversations with an alternative communication intervention is required to ascertain its’ relative value, and any unique contributions of this arts-based approach.
